# Effects of leukocytospermia on the outcomes of assisted reproductive technology

**DOI:** 10.1111/and.14403

**Published:** 2022-03-01

**Authors:** Xiaoyong Qiao, Rujun Zeng, Zhilan Yang, Liangzhi Xu, Qianhong Ma, Yezhou Yang, Yu Bai, Yihong Yang, Peng Bai

**Affiliations:** ^1^ 12530 Department of Obstetrics and Gynecology West China Second University Hospital Sichuan University Chengdu China; ^2^ 12530 Reproductive Endocrinology and Regulation Laboratory West China Second University Hospital Sichuan University Chengdu China; ^3^ The Joint Laboratory for Reproductive Medicine of Sichuan University The Chinese University of Hong Kong Chengdu China; ^4^ Key Laboratory of Birth Defects and Related Diseases of Women and Children (Sichuan University) Ministry of Education Chengdu China; ^5^ 12530 Department of Forensic Genetics West China School of Basic Medical Sciences & Forensic Medicine Sichuan University Chengdu China

**Keywords:** assisted reproductive technology, in vitro fertilization, intracytoplasmic sperm injection, leukocytospermia, split insemination

## Abstract

Leukocytospermia is one of the common causes of male infertility, and its effects on the clinical outcomes of assisted reproduction are controversial. There are no recommendations for the management of leukocytospermia in cases of assisted reproductive technology (ART). To investigate the impact of leukocytospermia on ART, we retrospectively compared the clinical outcomes in ART couples with or without leukocytospermia and further analysed the impact of the insemination method itself by split insemination treatment in ART couples with leukocytospermia. In this study, leukocytospermia was detected in 133 patients, namely 63 in the conventional in vitro fertilization (IVF) group, 38 in the intracytoplasmic sperm injection (ICSI) group and 32 in the split insemination group. Leukocytospermia has a negative influence on the parameters of semen samples; however, leukocytospermia did not affect the clinical outcomes of IVF or ICSI. Different insemination methods did not affect the fertilization, clinical pregnancy or live birth rates. In the split insemination study, no significant differences in clinical pregnancy and live birth rates between the IVF and ICSI groups were found; however, the numbers of two pronuclei (2PN), available embryos and good‐quality embryos in the ICSI group were higher than those in the IVF group. Leukocytospermia may be a risk factor affecting semen parameters, and more attention should be given to IVF insemination. Leukocytospermia has no significant negative effect on the outcomes of ART. ICSI may obtain better embryos than IVF, but it cannot improve the clinical pregnancy and live birth rates.

## 1 INTRODUCTION

Leukocytospermia is a common semen abnormality that accounts for 30% of male factor infertility (Brunner et al., 2019; Velez et al., 2021). According to the fifth World Health Organization (WHO) Laboratory Manual for the Examination and Processing of Human Semen, the presence of at least 1 × 10^6^ peroxidase‐positive cells/ml in a semen sample is considered leukocytospermia (also known as Pyospermia; Ford, 2010). Although it has been reported to increase infertility, its clinical significance and management are uncertain.

In theory, seminal leukocytes may play a role in stimulating toxic reactive oxygen species (ROS) which impair sperm cell functions and genital tract accessory glands. Excessive ROS induce lipid peroxidation by attacking the sperm plasma membranes, resulting in the loss of membrane function. Even so, to date, the clinical relationship between leukocytospermia and abnormal sperm parameters and function is unclear (Aitken, 2017; Henkel, 2011; Henkel et al., 2005; Lackner et al., 2008; Yilmaz et al., 2005). In particular, there is a correlation between leukocytospermia and reproductive tract infections (Henkel et al., 2021); therefore, it is difficult to clarify the exact effect of leukocytospermia on infertility. Due to the optimized processing of semen, there may be significant differences in its impact on natural pregnancy and assisted pregnancy.

Leukocytospermia, one of the common causes of male infertility, may adversely affect both sperm quality and fertilization (Lackner et al., 2010; Velez et al., 2021; Wolff et al., 1990). Many studies have investigated the relationships between semen quality and infertility (Castellini et al., 2020; Wolff et al., 1990; Yilmaz et al., 2005; Zorn et al., 2003). Some of them have shown that antimicrobial treatment of leukocytospermia may improve semen parameters and the natural pregnancy rate. However, whether leukocytospermia affects the outcomes of in vitro fertilization (IVF) and intracytoplasmic sperm injection (ICSI) is controversial (Castellini et al., 2020; Ricci et al., 2015; Yilmaz et al., 2005). Some studies have suggested that a large number of leukocytes in semen may affect the results of IVF and ICSI (Lackner et al., 2008; Yilmaz et al., 2005). However, Barraud‐Lange associated high leukocyte levels (more than 1 × 10^6^ leukocytes/ml) with an increased clinical pregnancy rate and suggested that seminal leukocytes are ‘good Samaritans’ for spermatozoa (Barraud‐Lange et al., 2011). Other studies also indicated that the role of leukocytospermia in poor IVF or ICSI results is uncertain (Castellini et al., 2020; Cavagna et al., 2012; Ricci et al., 2015). Due to the current lack of large‐sample randomized controlled trial (RCT) research, even for the general population preparing for pregnancy, there are great differences in the treatment and recommendations regarding leukocytospermia. Guidance regarding the clinical management of leukocytospermia in case of assisted reproduction is lacking (Brunner et al., 2019); therefore, we designed this study to assess whether leukocytospermia is a risk factor for IVF and ICSI outcomes to investigate the impact of leukocytospermia on assisted reproductive technology (ART). Due to the negative impact of leukocytospermia on semen parameters, the fertilization method itself may be adjusted accordingly, which may also be an important factor affecting the clinical outcome of ART. We further analysed the clinical outcomes of different insemination methods in cases of leukocytospermia to provide guidance for infertile men with leukocytospermia before ART.

## 2 MATERIALS AND METHODS

### 2.1 Study design

Patients were investigated at the Department of Reproduction of the West China Second University Hospital of Sichuan University between January 2017 and December 2020. All the patients signed informed consent forms, and this research was approved by an internal institutional review board. All the couples with female factor infertility and/or male factor infertility in their first cycles were included in this study. All the couples included met the following criteria: infertility with a need for ART, a first ART cycle, maternal age ≤40 years, a normal karyotype in both partners and no uterine defects, coagulation defects or thrombophilia defects.

The patients were divided into two groups (the leukocytospermia group and the non‐leukocytospermia group) according to the WHO definition of leukocytospermia as ≥1 × 10^6^ white blood cell (WBC)/ml semen. The couples in the non‐leukocytospermia group were selected as the control group, in which the male partner did not have leukocytospermia and the women had the same characteristics and fulfilled the following matching criteria: the same human chorionic gonadotropin (HCG) trigger and oocyte retrieval day and a comparable age (±1 year). If there were multiple patients who could serve as controls and meet the aforementioned matching criteria, the couple with comparable oocyte retrieval (±1) and comparable BMI was chosen. The data regarding IVF or ICSI were sorted into a form, and all the forms were summarized by one investigator. To avoid selection bias, the results of the potential control couples were hidden until the couples were selected. To investigate whether the insemination method affected the clinical outcomes, we specifically designed an additional split insemination group, in which all the couples with sibling oocytes were treated with both conventional IVF and ICSI. To carry out split insemination, the semen needed to meet the requirements for conventional IVF. Mature oocytes were randomly assigned to the IVF group (conventional IVF group) or the ICSI group (ICSI group). If the number of oocytes was odd, one remaining oocyte was assigned to the ICSI group.

### 2.2 Semen preparation and analysis

The semen samples were collected in sterile collection cups after a sexual abstinence period of 2 to 7 days. The samples were prepared and analysed for sperm concentration, forward progressive motility and total motility rate after semen preparation according to WHO criteria (WHO, 2010). In our study, ICSI was performed if any of the following occurred in the semen analysis: the seminal concentration was less than 10 × 10^6^/L, the progressive sperm motility was less than 32%, or the percentages of spermatozoa with normal morphology were less than 1%; otherwise, conventional IVF insemination or split insemination was selected. In brief, the semen samples were prepared by a two‐layer (90% and 45%) discontinuous Pure Sperm (Nidacon, Goteborg, Sweden) gradient technique. One millilitre of semen was added to the top of two layers in a centrifuge tube and then centrifuged at 400 *g* for 20 min. Then, the supernatant was removed, and the pellet at the bottom was suspended with 1 ml medium and centrifuged at 500 *g* for 10 min again. The sperm leukocyte concentration was examined by the leukocytospermia peroxidase test (LCPT), which was performed using a commercially available kit (Anhui Anke Biotechnology Co., Ltd). Following the instructions to prepare the working solution by mixing hydrogen peroxide and the benzidine/cyanamide solution, 100 μl of the semen sample was added, the mixture was mixed for 30 min, the mixture was dripped into a cell counting pool, and the cells were counted under a phase contrast microscope. Brown‐stained cells were regarded as peroxidase positive, while the pink and unstained cells were regarded as peroxidase negative.

### 2.3 Ovarian stimulation and embryo development

All of the patients received a gonadotropin‐releasing hormone (GnRH) agonist protocol or GnRH antagonist protocol. In the GnRH agonist long protocol, the patients were processed for pituitary downregulation during the middle luteal phase of the previous cycle with triptorelin acetate at a dose of 0.1 mg/48 h (Ipsen Pharma Biotech). After confirmation of pituitary downregulation by ultrasonography and basic hormone levels, the administration of recombinant follicle‐stimulating hormone (FSH; Merck KGaA) was started at a dose of 150–225 IU (the initial gonadotropin dose was dependent on the patient's age and BMI). After 4 days of fixed‐dose stimulation, the development of follicles was assessed and optimally adjusted based on the number and size of the developing follicles. In the antagonist protocol, evaluation was conducted by ultrasound and detection of basic hormone levels on Day 3 of the menstrual period. Then, medication was initiated with recombinant FSH as described above in the GnRH agonist long protocol, and the GnRH antagonist cetrorelix (0.25 mg, Cetrotide, Merck‐Serono) was administered daily from Day 6 of the stimulation cycle to the day of HCG trigger. When two or more follicles reached a diameter of 17 mm, oocyte retrieval was scheduled 36 h after 5000 or 10000 IU of HCG (Livzon) injection. Fertilization was evaluated 16–18 h after conventional insemination or ICSI. On Day 3, the embryos from both the ICSI and/or IVF groups were transferred. The ART clinical outcomes in the non‐leukocytospermic and leukocytospermic couples were compared.

### 2.4 Statistical analysis

The endpoints of this investigation included the semen parameters pre‐ and postsemen processing for morphological examination and the fertilization, clinical pregnancy, miscarriage and live birth rates. Fertilization was defined as zygotes containing one,two or three pronuclei after IVF or ICSI insermination. Zygotes containing two pronuclei was defined as 2PN in particular. Clinical pregnancy was defined as a gestational sac with a heartbeat detected by transvaginal ultrasound 4–6 weeks after embryo transfer. Miscarriage was defined as the termination of the pregnancy at no more than 28 weeks of gestation. Live birth was defined as delivery of a live baby with ≥28 weeks. Statistical analysis was conducted with the Statistical Package for the Social Sciences (Ver. 23; SPSS Inc.) software. Continuous data were confirmed for normal distribution by the Kolmogorov–Smirnov test, and data are reported as the mean ± SD. Categorical data are presented as frequencies and/or percentages. Student's *t*‐tests were used for normally distributed variables, while the Mann–Whitney *U*‐test was used for non‐normally distributed variables. Comparison among more than three groups was made by analysis of variance (ANOVA) test or chi‐square tests. Chi‐square tests or Fisher's exact tests were used to analyse the differences between percentages for categorical data. All hypothesis testing was two‐tailed, and a *p* value < 0.05 was considered statistically significant.

## 3 RESULTS

Leukocytospermia was detected in 133 patients, namely 63 patient in the conventional IVF insemination group, 38 in the ICSI group and 32 in the split insemination group. The main clinical characteristics and outcomes, namely the semen parameters and rates of fertilization, clinical pregnancy, miscarriage and live birth, in the IVF and ICSI groups with or without leukocytospermia are shown in Tables 1 and 2. The clinical characteristics and results of all the patients with leukocytospermia are shown in Table 3. The comparison between IVF and ICSI in the split insemination leukocytospermia group is listed in Table 4.

**TABLE 1:** Comparison between the non‐leukocytospermia and leukocytospermia groups in IVF

Variable	Non‐leukocytospermia (*n* = 63)	Leukocytospermia (*n* = 63)	*p* value
Female age (year)	30.2 ± 3.8	30.70 ± 3.9	0.43^a^
Female BMI (kg/m^2^)	21.98 ± 2.53	21.37 ± 2.99	0.22^a^
AMH (ng/ml)	3.77 ± 2.53	3.89 ± 2.23	0.76^a^
Ovarian stimulation protocol			
GnRH agonist long protocol (*n*)	33	35	0.72^b^
GnRH antagonist protocol (*n*)	30	28	0.72^b^
Sperm concentration (×10^6^/ml)	70.8 ± 24.6	55.0 ± 24.1	0.01^a^
Sperm concentration (after; ×10^6^/ml)	87.0 ± 26.8	70.0 ± 25.6	0.01^a^
Forword progressive (×10^6^/ml)	32.6 ± 13.4	23.8 ± 11.8	0.01^a^
Forword progressive (after; ×10^6^/ml)	63.6 ± 22.6	49.2 ± 20.6	0.01^a^
Spermatozoa with normal morphology (%)	5.63 ± 3.85	4.26 ± 3.56	0.12^a^
Oocyte retrieval (*n*)	10.05 ± 4.55	9.67 ± 5.08	0.66^a^
Mature oocytes (*n*)	8.16 ± 3.94	8.02 ± 4.81	0.86^a^
2PN (*n*)	6.2 ± 3.1	5.9 ± 4.2	0.65^a^
1PN (%)#	0.00 (0.00–0.11)	0.00 (0.00–0.13)	0.04^#^
3PN (%)#	0.00 (00.00–0.08)	0.00 (0.00–0.00)	0.41^#^
Fertilization (%)	0.74 ± 0.16	0.72 ± 0.19	0.66^a^
Good quality embryos (*n*)	1.73 ± 1.67	1.59 ± 1.76	0.48^a^
Available embryo (*n*)	2.97 ± 2.00	2.68 ± 2.47	0.18^a^
Pregnancy/cycle (%)	52.3% (33/63)	46.0% (29/63)	0.48^b^
Miscarriage (%)	12.1% (4/33)	17.2% (5/29)	0.72^b^
live birth/cycle (%)	46.0% (29/63)	38.1% (24/63)	0.41^b^

Abbreviation: 2PN, zygotes containing two pronuclei.

after:after the semen was processed and optimized by gradient centrifugation.

^a^ Student's *t*‐test.

^b^ Chi‐square test or Fisher's exact test.

# Mann–Whitney *U*‐test.

* Results are presented as median with 25th, 75th quartiles.

**TABLE 2:** Comparison between the non‐leukocytospermia and leukocytospermia groups in ICSI

Variable	Non‐leukocytospermia (*n* = 38)	Leukocytospermia (*n* = 38)	*p* value
Female age (year)	29.8 ± 3.6	29.3 ± 4.4	0.94^*^
Female BMI (kg/m^2^)	21.5 ± 2.83	21.7 ± 2.46	0.70^*^
AMH (ng/ml)	4.04 ± 1.34	4.23 ± 1.61	0.57^*^
Ovarian stimulation protocol			
GnRH agonist long protocol (*n*)	25	24	0.81^a^
GnRH antagonist protocol (*n*)	13	14	0.81^a^
Sperm concentration (×10^6^/ml)	33.8 ± 33.3	25.0 ± 24.6	0.19^*^
Sperm concentration (after; ×10^6^/ml)	45.6 ± 38.2	35.5 ± 29.7	0.20^*^
Forward progressive (×10^6^/ml)	13.0 ± 16.9	6.5 ± 8.9	0.04^*^
Forward progressive (after; ×10^6^/ml)	28.2 ± 31.1	16.0 ± 17.8	0.04^*^
Spermatozoa with normal morphology (%)	2.46 ± 2.13	2.04 ± 1.88	0.42^*^
Oocyte retrieval (*n*)	10.11 ± 3.55	10.05 ± 3.47	0.95^*^
Mature oocytes (*n*)	7.2 ± 3.2	7.6 ± 3.3	0.57^*^
2PN (*n*)	5.5 ± 2.7	5.5 ± 3.1	0.95^*^
Fertilization (%)	0.85 ± 0.16	0.87 ± 0.13	0.46^*^
Good quality embryos (*n*)	1.46 ± 1.26	1.92±1.50	0.15^*^
Available embryos (*n*)	2.76 ± 1.27	3.3 ± 2.0	0.20^*^
Pregnancy/cycle (%)	50.0% (19/38)	47.4% (18/38)	0.82^a^
Miscarriage (%)	10.5% (2/19)	16.6% (3/18)	0.66^a^
live birth/cycle (%)	44.7% (17/38)	39.5% (15/38)	0.64^a^

Abbreviation: 2PN, zygotes containing two pronuclei.

after:after the semen was processed and optimized by gradient centrifugation.

^a^ Student's *t*‐test.

^b^ Chi‐square test or Fisher's exact test.

**TABLE 3:** Comparison of different insemination treatments in leukocytospermia

Variable	Leukocytospermia (IVF *n* = 63)	Leukocytospermia (ICSI *n* = 38)	Leukocytospermia (Half *n* = 32)	*p* value
Female age (year)	30.7 ± 3.9	29.3 ± 4.4	31.0 ± 4.8	0.17^b^
Female BMI (kg/m^2^)	21.37 ± 2.99	21.70 ± 2.46	21.40 ± 3.35	0.83^b^
AMH (ng/ml)	3.89 ± 2.23	4.23 ± 1.61	4.12 ± 2.70	0.04^b^
Sperm concentration (×10^6^/ml)	55.0 ± 24.1	25.0 ± 24.6	60.9 ± 27.5	0.01^b^
Sperm concentration (after; ×10^6^/ml)	70.0 ± 25.6	35.5 ± 29.7	81.1 ± 34.1	0.01^b^
Forward progressive (×10^6^/ml)	23.8 ± 11.8	6.5 ± 8.9	39.0 ± 9.5	0.01^b^
Forward progressive (after; ×10^6^/ml)	49.2 ± 20.6	16.0 ± 17.8	63.6 ± 11.7	0.01^b^
Spermatozoa with normal morphology (%)	4.26 ± 3.56	2.04 ± 1.88	4.43 ± 3.28	0.01^b^
Oocyte retrieval (*n*)	9.67 ± 5.08	10.05 ± 3.47	13.16 ± 5.5	0.03^b^
Mature oocytes (*n*)	8.02 ± 4.81	7.60 ± 3.3	10.69 ± 4.50	0.01^b^
2PN (*n*)	5.90 ± 4.2	5.50 ± 3.1	8.03 ± 4.08	0.01^b^
Fertilization (%)	0.72 ± 0.19	0.87 ± 0.13	0.68 ± 0.24	0.01^b^
Good quality embryos (*n*)	1.59 ± 1.76	1.92 ± 1.50	3.22 ± 2.66	0.01^b^
Available embryos (*n*)	2.68 ± 2.47	3.30 ± 2.0	5.10 ± 3.28	0.01^b^
Pregnancy/cycle (%)	46.0% (29/63)	47.4% (18/38)	56.5% (26/46)	0.53^a^
Miscarriage (%)	17.2% (5/29)	16.6% (3/18)	7.7% (2/26)	0.57^a^
live birth/cycle (%)	38.1% (24/63)	39.5% (15/38)	52.2% (24/46)	0.30^a^

Note

Half: split insemination treatment (conventional IVF and ICSI) on sibling oocytes

2PN: zygotes containing two pronuclei.

after:after the semen was processed and optimized by gradient centrifugation

^a^ Analysis of variance (ANOVA) test.

^b^ Chi‐square test or Fisher's exact test.

**TABLE 4:** Comparison between the IVF and ICSI in split insemination leukocytospermia groups

Variable	Total (*n* = 32)	IVF (*n* = 32)	ICSI (*n* = 32)	*p* value
Oocyte retrieval (*n*)	13.16 ± 5.5	6.47 ± 2.78	6.53 ± 2.54	0.601^b^
Mature oocytes (*n*)	10.69 ± 4.50	5.22 ± 2.30	5.44 ± 2.12	0.304^b^
2PN (*n*)	8.03 ± 4.08	3.63 ± 2.48	4.59 ± 2.18	0.006^b^
Fertilization (%)	0.68 ± 0.24	0.63 ± 0.33	0.72 ± 0.22	0.085^b^
Available embryos (*n*)	5.1 ± 3.28	2.16 ± 1.87	3.0 ± 1.81	0.007^b^
Good quality embryos (*n*)	3.22 ± 2.66	1.34 ± 1.03	1.97 ± 1.53	0.005^b^
Pregnancy/cycle (%)	56.5% (26/46)	50% (11/22)	62.5% (15/24)	0.393^a^
Miscarriage (%)	7.7% (2/26)	9.1% (1/11)	6.7% (1/15)	1.000^a^
Live birth/cycle (%)	52.2% (24/46)	45.5% (10/22)	58.3% (14/24)	0.382^a^

Abbreviation: 2PN: zygotes containing two pronuclei.

^a^ Student's *t*‐test.

^b^ Chi‐square tests or Fisher's exact test.

### 3.1 Effects on IVF outcome

During this study period, 63 patients with leukocytospermia who met the inclusion criteria were analysed. Sixty‐three cases in the control group were matched and included according to a ratio of 1:1. A total of 126 patients underwent conventional IVF insemination. The baseline characteristics are presented in Table 1. The mean ages of patients with or without leukocytospermia were similar in both groups. There were no differences in female BMI, anti‐Müllerian hormone (AMH) level, Ovarian stimulation protocol or the number of mature oocytes. However, the data showed that leukocytospermia had a negative effect on the quality of the semen samples. The sperm concentrations and forward progressive motility rates were lower in the leukocytospermic group than in the non‐leukocytospermic group before and after semen preparation. There were significant differences in forward progressive motility rates and sperm concentrations between the groups (*p* < 0.05; Table 1). The proportion of spermatozoa with normal morphology was not statistically different between the two groups. The association of leukocytospermia with semen quality was not affected after adjusting for factors such as age, smoking status, varicose veins and abstinence period by multiple linear regression model analysis. In addition, there was a higher 1PN rate in the leukocytospermic group than in the non‐leukocytospermic group. To evaluate the cycles outcomes, we used the rates of fertilization, the number of available embryos, the number of good‐quality embryos, the clinical pregnancy rate, the miscarriage rate and the live birth rate, all the parameters above were similar between the groups as shown in Table 1.

### 3.2 Effects on ICSI outcome

In this part, 38 patients with leukocytospermia and 38 controls without leukocytospermia underwent ICSI. The distributions of the clinical characteristics observed for the ICSI were comparable between the groups (*p* > 0.05) as summarized in Table 2. There was no significant difference in the baseline characteristics in the leukocytospermic group compared with the non‐leukocytospermic group, except for the lower forward progressive motility rates in the leukocytospermic group compared with the non‐leukocytospermic group before and after semen preparation. There was no difference in semen concentration and the proportion of spermatozoa with normal morphology between the groups, and no statistical difference in available embryos and good‐quality embryos between the two groups. In addition, the rates of fertilization, clinical pregnancy, miscarriage and live birth were not impacted by the presence of leucocytes between the groups as listed in Table 2 (*p* > 0.05).

### 3.3 Effects on split insemination outcome

Previous research has shown that leukocytospermia is related to the decreased quality of semen samples. Worse semen parameters may change the method of insemination. To further clarify whether the method of fertilization affects the clinical outcome of leukocyte semen, we further analysed the outcome of ART with split insemination. We treated 32 couples with split insemination, and our results showed that there were no differences in the fertilization, clinical pregnancy or live birth rates among the IVF, ICSI and split insemination groups as presented in Table 3. In addition, the oocyte retrieval, number of mature oocytes and fertilization rate were also similar between the IVF and ICSI groups in the split insemination study. However, the number of 2PN (4.59 ± 2.18 versus 3.63 ± 2.48, *p* = 0.006), available embryos (3.0 ± 1.81 versus 2.16 ± 1.87, *p* = 0.007) and good‐quality embryos (1.97 ± 1.53 versus 1.34 ± 1.03, *p* = 0.005) in the ICSI group were greater than those in the IVF group (Table 4). No significant differences in the clinical pregnancy and live birth rates between the IVF and ICSI groups were found in the split insemination group.

## 4 DISCUSSION

To date, the clinical significance of leukocytospermia and its effects on IVF and ICSI outcomes are controversial. There are few reports analysing the effects of leukocytospermia in cases of ART. In our study, we analysed the effect of leukocytospermia on semen parameters and clinical outcomes in ART.

Our research has shown that there are significantly adverse impacts on sperm concentration and forward progressive motility rate in leukocytospermia patients compared to non‐leukocytospermia patients (Eldamnhoury et al., 2018). The poor sperm quality associated with leukocytospermia may be the outcome of ROS and inflammatory mediators released by peroxidase‐positive leukocytes. Activated leukocytes produce more ROS than ROS‐producing spermatozoa (Wolff et al., 1990). In vitro, ROS have been univocally demonstrated to be highly toxic. The high polyunsaturated fatty acid content of spermatozoa membrane increases their susceptibility to lipid peroxidation by ROS, which impairs tail motion (Henkel, 2011). Many studies have shown that an increased number of leukocytes in seminal fluid are associated with worse sperm parameters. Semen samples with elevated WBC counts show significant decreases in total sperm number, sperm motility index and total number of motile sperm (Wolff et al., 1990). Diemer observed a reduction in the progressive motile sperm rate in vitro after incubation with leucocytes (Diemer et al., 1994). These findings correlate well with our results, which demonstrated decreased sperm concentration and progressive motility.

Many studies have indicated that leukocytospermia is associated with poor sperm parameters, including sperm concentration, forward progressive motility and morphology and fertilization (Castellini et al., 2020). Some earlier studies have suggested the negative effects of leukocytospermia on fertilization (Maruyama et al., 1985; Talbert et al., 1987; Wolff et al., 1990). In 1985, Maruyama first reported that leukocytospermia have a negative effect on the heterologous sperm penetration assay (SPA) in vitro (Maruyama et al., 1985). The results showed that the increased numbers of WBCs in semen were related to poor SPA scores and that administration of antibiotics to men with leukocytospermia could increase the pregnancy rate during the following year. This result seems to be consistent with the findings that leukocytospermia has consequent adverse effects on sperm fertilizing capacity (Omu et al., 1999). Leukocytes have been reported to negatively influence fertilization in IVF cycles (Aitken et al., 1994; Lackner et al., 2008). Aziz et al. reported that leukocytospermia is associated with sperm deformity index scores, acrosomal damage, midpiece defects and tail deformities (Aziz et al., 2004). These studies show the negative effects of leukocytospermia on fertilization.

In our study, we found that the fertilization rate was similar in the leukocytospermic group and non‐leukocytospermic group, which is consistent with many studies published in the latest decade or more. However, the exact effects of leukocytospermia on fertilization in cases of ART are still unclear. Some authors have suggested that leukocytospermia does not hinder any sperm fertilization functions (Barraud‐Lange et al., 2011; Yanushpolsky et al., 1996), and even would have beneficial effects in supporting the induction of the sperm acrosome reaction. One possible explanation for this contradiction is that some studies assessed only a small number of leukocytospermic semen samples. Another possible explanation is that the method and criteria of diagnosing leukocytospermia in some previous studies were different from those in a recent study. For example, Peroxidase staining is not regarded as the gold standard but the standard method for detecting semen leukocytes (Velez et al., 2021). However, Ricci et al. revealed that leukocytospermia does not have significant negative effects on IVF or ICSI outcomes by using the method of flow cytometry to evaluate the effects of leukocytospermia even after adjusting the leukocytospermia cut‐off from 0.2 × 10^6^ WBCs/ml to 2 × 10^6^ WBCs/ml (Ricci et al., 2015). There was no difference in the fertilization in couples with or without leukocytospermia in ART (Ricci et al., 2015).

There are differences between experimental in vitro and in vivo data on leukocytospermia. One of the possible reasons is that the most frequently used methods for sperm preparation, including density‐gradient centrifugation and swim‐up in ART, eliminate most of the harmful substances in sperm and improve the concentration and quality of spermatozoa. It reduces the negative impact of leukocytospermia on insemination parameters. Another reason is the proper adjustment of insemination methods after accurate evaluation of semen in the ART laboratory before insemination. When the quantity or quality of semen is poor, the use of ICSI may further reduce the risk of abnormal insemination. In particular, the use of ICSI has increased dramatically and become the most frequently used method of insemination in recent years. The adjustment and changes of insemination methods may also be one of the factors that weakens the influence of leukocytospermia on fertilization.

Compared with the question of fertilization rate, whether leukocytospermia affects the clinical outcome of ART seems more confusing and difficult to answer. Yilmaz et al. found that the rates of fertilization and embryo development were significantly lower in the leukocytospermic group than in the control group (Yilmaz et al., 2005). However, most studies have suggested that there is no significant difference in the clinical outcome of leukocytospermia compared with non‐ leukocytospermia. A large follow‐up study over 1,900 couples indicated that a higher WBC count in semen is associated with a higher fertilization rate, cleavage rate and clinical pregnancy rate, although leukocytospermia is associated with a higher rates of miscarriage and ectopic pregnancy than non‐leukocytospermia (Barraud‐Lange et al., 2011). These results lead to a new question whether it is necessary to treat leukocytospermia if the couple is scheduled for IVF. Our result indicated that the clinical outcome of leukocytospermia is not better than that of non‐leukocytospermia. There was no statistical difference between the two groups. Similar to the lack of difference in fertilization mentioned above, it is difficult to clarify whether this undifferentiated change is due to the optimization of semen, the different requirements for semen for ART or the choice of insemination methods by embryologist, which have improved the clinical outcome. In particular, existing studies on leukocytospermia patients compared to non‐leukocytospermia patients have shown that the use of IVF or ICSI has no significant effect on clinical outcomes (Castellini et al., 2020); therefore, laboratory physicians may use IVF insemination more when the quality of sperm is good, while ICSI will commonly applied when the sperm are abnormal and may possibly affect conventional IVF fertilization.

In our study, we retrospectively compared the clinical outcomes of split insemination (conventional IVF and ICSI). Compared with traditional IVF and ICSI, there was no significant difference in the fertilization rate, clinical pregnancy rate and live birth rate in split semination in the leukocytospermia group. Additionally, in the study of the insemination method, there was also no difference in the fertilization rate, clinical pregnancy rate or live birth rate between conventional IVF and ICSI in separate fertilization in the leukocytospermia group. Cavagnade reported that leukocytospermia may not have a negative effect on the outcomes of ICSI and intracytoplasmic morphologically selected sperm injection cycle (Cavagna et al., 2012). However, the number of 2PN, available embryos and good‐quality embryos in ICSI was higher than that in IVF in our study, and whether the difference in split fertilization is related to semen optimization or to changing fertilization methods that further reduce the impact of WBC still needs more research. Tannus reported that ICSI does not have additional benefits in couples with advanced maternal age and without male factor infertility (Tannus et al., 2017), while Chamayou reported that more blastocysts are obtained from fewer oocytes in ICSI compared to IVF for non‐severe male factor infertility (Chamayou et al., 2021). ICSI was more efficient in producing more embryos than IVF in the leukocytospermia group in our study. For patients, especially for patients with fewer MII oocytes, more embryos from ICSI may increase the probability of pregnancy and live birth. Whether this difference in embryonic development between conventional IVF and ICSI in our study will affect the cumulative clinical pregnancy rate or the cumulative live birth rate requires further research. From the present study, fertilization, clinical outcomes such as clinical pregnancy rate and live birth rate are comparable in split insemination cycles. Because more embryos are produced in ICSI than in IVF in cases of leukocytospermia, conventional IVF and split insemination cycles need to be carefully proposed according to semen parameters and oocyte numbers, ICSI may better improve the impact of leukocytospermia.

In summary, our research, from a new perspective, comprehensively analysed and compared the effects of leukocytospermia on ART. First, compared with non‐leukocytospermia, leukocytospermia did not affect the main clinical outcome of IVF. Different insemination methods also did not affect the rates of fertilization, clinical pregnancy and live birth. However, ICSI may obtain more embryos when the sperm meet the requirement of conventional IVF insemination. Nevertheless, there are some limitations to this study. Our research is a retrospective analysis with a limited sample size. In addition, we used standard methods for the diagnosis of WBCs rather than more accurate methods, such as multiparameter flow cytometric analysis. Third, we did not analyse the status of reproductive tract infections.

## 5 CONCLUSION

Leukocytospermia may be a risk factor affecting semen parameters, and more attention should be given to IVF insemination. Leukocytospermia has no significant negative effect on ART. ICSI may obtain better embryos than IVF, but it cannot improve the clinical pregnancy and live birth rates.

## CONFLICT OF INTEREST

The authors have no interests to disclose.

## AUTHORS’ CONTRIBUTIONS

All the authors approved the submitted version of this paper and gave substantial contributions to the research design and critically revising the paper. In particular, Xiaoyong Qiao involved in paper writing, concept and research design, data analysis, interpretation, manuscript draft and manuscript revision. Rujun Zeng and Qianhong Ma involved in material preparation and statistical analysis. Zhilan Yang involved in patient selection and data collection. Liangzhi Xu involved in data interpretation and statistical analysis. Yezhou Yang involved in material preparation and critically revising the paper. Yu Bai and Peng Bai involved in manuscript draft and manuscript revision. Yihong Yang involved in paper writing, research design, material preparation and data analysis.

## ETHICS APPROVAL

This retrospective study involving human participants was in accordance with the ethical standards and the 1964 Helsinki Declaration and its later amendments or comparable ethical standards. Our study had got granted exemption from the Medical Ethics Committee of West China Second University Hospital of Sichuan University.

## INFORMED CONSENT

Written informed consent was obtained from individual or guardian participants.

## Data Availability

All data generated or analysed during this study are included in this published article.
